# Fight against Disease

**Published:** 1914-01

**Authors:** 


### Fight Against Disease.

Uncle Sam's health patrol is catching its breath on New Year's Day in anticipation of the biggest year's work it has ever undertaken. The summary for the twelve months of its work contains a record of heroic fights against disease, opposition to the invasion of foreign maladies and excursions into new fields of medical exploration which had no equal in the history of the organization.

Experts working under the direction of Surgeon General Rupert Blue and Assistant Surgeon General Rucker, have, during the last year, covered every nook and corner of the country, routing out unknown diseases, cleaning up infested places, teaching communities and individuals who guard against disease, and protecting the American public against diseased immigrants, unhealthful surroundings, infected rats and polluted drinking water.

From the mountains of Kentucky, where two field hospitals and dispensaries are teaching the people how to combat pellagra, to the water fronts of San Francisco and Seattle, where Federal experts are helping State authorities in their fight to prevent the carrying of plague germs by rats and squirrels, the public health service has pushed its activities into many unusual fields.

Much of the research work for the service is done in the hygienic laboratory in Washington. In the search for the means by which infantile paralysis is transmitted, the experts there succeeded during the last year in carrying germs from one monkey to another by the bite of a stable fly. But where this succeeded once, it failed on all other attempts and the public health service is still working on the problem of coping with this disease.

Pollution of the rivers and other dangers to communities close to them is under examination now in the valleys of the Missouri, Mississippi, Ohio and Potomac. A comprehensive investigation of pellagra is being directed from Savannah, and malaria is being studied at the Mobile Marine Hospital.

Practically every phase of sanitation is being studied and taught by the public health service officials as fast as funds permit. During the last year each expert of the service, no matter on what work he might be detailed, has been acting as a traveling inspector of railway trains, and vessels and reporting any violations of the interstate regulations governing drinking cups, pure water, roller towels and the like.

Over 39,000 Indians on reservations in twenty-five States were examined under direction of the public health service during the last year to determine the prevalence of trachoma and consumption. More than 22 per cent of the Indians were found to be suffering from trachoma, and drastic measures have been recommended by the health officers to meet the situation.

---

Melted vaseline is an important adjunct in the transfusion paraphernalia. Frequently applied at the site of juncture, it minimizes any tendency to clot formation.—*American Journay of Surgery.*

